# Repurposing diclofenac for combination therapy: targeting the Warburg effect to enhance breast cancer treatment

**DOI:** 10.55730/1300-0144.6373

**Published:** 2026-03-07

**Authors:** Gonca TUNA, Elif ERTÜRK, Ayşen SAĞNAK, Ferda ARI

**Affiliations:** 1 Department of Biology, Science and Art Faculty, Bursa Uludag University, Bursa, Turkiye; 2 Medical Laboratory Techniques Program, Vocational School of Health Services, Bursa Uludag University, Bursa, Turkiye

**Keywords:** Diclofenac, glycolysis, breast cancer, triosephosphate isomerase

## Abstract

**Background/aim:**

Diclofenac (Diclo), a widely used nonsteroidal antiinflammatory drug, has emerged as a potential candidate for drug repurposing in oncology, with reported anticancer effects extending beyond cyclooxygenase (COX) inhibition, including modulation of tumor metabolism. This study investigated the antiproliferative and glycolysis-associated effects of Diclo in MCF-7 breast cancer cells, alone and in combination with docetaxel (Doc) or 5-fluorouracil (5-FU).

**Materials and methods:**

Cell viability was assessed using the sulforhodamine B (SRB) assay. To evaluate metabolic alterations, glucose uptake and lactate release were quantified following treatment. In addition, Triosephosphate Isomerase (TPI) protein levels and Lactate Dehydrogenase (LDH) activity were measured to explore potential modulation of glycolytic pathways.

**Results:**

Diclo reduced MCF-7 viability in a dose-dependent manner (IC_50_: 78.37 μg/mL). Combining Diclo with Doc or 5-FU enhanced cytotoxicity compared with single agents. Although Diclo alone did not significantly change glucose uptake, Diclo+Doc and Diclo+5-FU significantly decreased glucose uptake, and lactate release was reduced, particularly in Diclo-containing treatments. Diclo decreased TPI levels, and Diclo-based combinations further reduced TPI. LDH activity decreased with Diclo, while certain combinations produced divergent LDH responses, suggesting treatment-dependent effects on lactate metabolism and/or cellular stress.

**Conclusion:**

Diclo potentiated the cytotoxic effects of Doc and 5-FU and was associated with reduced glycolytic readouts and decreased TPI in MCF-7 cells, supporting a potential role for Diclo as a metabolic modulator in breast cancer combination therapy.

## 1. Introduction

Cancer, characterized by uncontrolled cell growth and division, is one of the leading causes of death worldwide, with findings suggesting that the number of new cancer cases could reach 35.3 million annually by 2050 [1,2]. With an expected 2.3 million new cases globally, breast cancer is the second most frequent kind of cancer after lung cancer^1^. Research aimed at devoloping anticancer agents has grown in importance due to the seriousness of cancer as a worldwide health concern. Among anticancer drug studies, research targeting energy metabolism occupies a substantial area. Cancer metabolism is considered a key component of cellular transformation, and notable examples support the clinical applicability of targeting metabolism [3].

In order to meet the energy requirements for aberrant cell growth and division, cancer cells rewire their energy metabolism [4]. In normal cells, under aerobic conditions, glucose is first converted to pyruvate via glycolysis in the cytosol and then further metabolized into carbon dioxide through oxidative phosphorylation (OXPHOS) in the mitochondria to generate energy. Under anaerobic conditions, glycolysis is predominantly utilized, leading to the production of lactate. Cancer cells, however, rewire their glucose metabolism by favoring glycolysis even in the presence of oxygen, converting glucose into lactate. The “Warburg Effect” or “aerobic glycolysis” are terms used to describe this process [5,6]. Therefore, blocking this route is seen to be a promising strategy for cancer treatment and the development of anticancer drugs because glycolysis is essential to the energy metabolism of cancer. Accordingly, various molecules with inhibitory effects on different glycolytic enzymes are being investigated.

Diclofenac (Diclo) is a common nonsteroidal antiinflammatory drug (NSAID) that alleviates symptoms such as inflammation, pain, and fever. Global sales of Diclo were reported to reach €95 million between 2016 and 2019^2^. The primary mechanism of action of NSAIDs is known to involve the inhibition of cyclooxygenases (COX), which play a role in pain and inflammation. Additionally, it has been demonstrated that NSAIDs, including Diclo, suppress tumor formation and cancer progression through various cellular mechanisms [7–9]. It is known that Diclo may have anticancer effects in various cancer types, including neuroblastoma, osteoblastoma, glioma, colorectal cancer, fibrosarcoma, lung cancer, and pancreatic cancer. Its effects have been primarily studied concerning apoptosis and cell cycle mechanisms [10]. In addition to Diclo treatment alone, its use in combination with other drugs has also demonstrated promising results [11,12]. Given the importance of targeting energy metabolism in cancer cells, studies investigating the effects of Diclo on glucose metabolism have gained prominence. For example, research on the COX-independent effects of Diclo has reported a significant reduction in Myc expression in melanoma and leukemia cells, disrupting cell proliferation by modulating glucose metabolism [13]. By altering glucose metabolism, Diclo has also been demonstrated to inhibit the proliferation of triple-negative breast cancer cells [14].

Mechanistically, Diclo is expected to influence cancer cell survival through both COX-dependent and COX-independent pathways. COX-2 inhibition can reduce prostaglandin E2 (PGE2) signaling, which is linked to prosurvival and proproliferative cascades (e.g., PI3K/AKT and ERK/MAPK) and may indirectly reshape glucose metabolism [9]. In parallel, COX-independent actions of Diclo have been reported to suppress MYC-driven metabolic programs, including glucose transport and lactate-associated genes, thereby impacting glycolytic flux [13,14]. Based on these reports, we hypothesized that Diclo could act as a metabolic modulator and enhance the efficacy of cytotoxic agents by attenuating glycolysis-related endpoints.

Additionally, investigating the anticancer activity of Diclo, in addition to its analgesic and antipyretic effects, holds a significant position within the scope of drug repurposing studies. Drug repurposing refers to identifying new therapeutic uses for clinically approved drugs beyond their original indications. Since the development of new drugs is a highly complex and costly process, determining new effects of drugs with already known pharmacological properties and established clinical use offers a lower-cost, faster, and less risky alternative [15]. In this study, the antiglycolytic effects of Diclo treatment alone and in combination with chemotherapy drugs Docetaxel (Doc) and 5-Fluorouracil (5-FU) in breast cancer have been investigated for the first time. Although 5-FU has historically been used in breast cancer, it is currently less frequently included in routine clinical regimens compared with modern anthracycline-/taxane-based protocols and subtype-directed therapies. Therefore, 5-FU was used in this study primarily as a well-characterized in vitro cytotoxic reference to contextualize Diclo-related antiproliferative and metabolic effects.

## 2. Material and methods

### 2.1. Cell culture

RPMI 1640 medium (Gibco, USA) supplemented with 2 mM L-glutamine (Gibco, USA), 10% FBS, and 100 U/mL penicillin + 100 μg/mL streptomycin (Gibco, USA) was used to cultured the human breast cancer cell line (MCF-7) at 37 °C and 5% CO_2_ until the number of cells was sufficient for the experiments. MCF-7 is a widely used ER/PR positive (lumen type) breast cancer model for studying hormone-sensitive biological processes and metabolic adaptations; it also exhibits a metabolic phenotype based on oxidative phosphorylation, and was therefore selected to test whether Diclo can regulate glycolytic outcomes in a clinically prevalent breast cancer subtype [16, 17].

### 2.2. Diclo treatment and sulforhodamine B (SRB) viability analysis

To evaluate the effect of Diclo on the viability of MCF-7 cells, the SRB viability assay was performed. Diclo was obtained in ampoules containing a 75 mg/3 mL injectable solution (#Abdi İbrahim, Türkiye). It was prepared in concentrations ranging from 0.39 to 400 μg/mL in the culture medium and applied to cells seeded at a density of 5×10^3^ cells/well in 96-well cell culture plates for 48 h. At the end of the incubation period, the SRB assay was conducted [18].

### 2.3. Combination treatment in MCF-7 cells

To assess the anticancer effects of combination treatments, MCF-7 cells were seeded into 96-well plate for viability test. Diclo at concentrations of 25, 50, and 100 μg/mL was combined with 5-FU (0.35–22.5 μg/mL) and Doc (0.17–11.3 μg/mL) and applied to the cells. We used Diclo doses below, near, and above the IC_50_ (25, 50, and 100 μg/mL) to capture potential nonlinear interaction patterns and to evaluate whether metabolic modulation and cytotoxic potentiation occur at sub-IC_50_ exposures. Following 48 h of incubation, cell viability was evaluated using the SRB assay.

### 2.4. Measurement of glucose uptake

Changes in glucose levels in MCF-7 cells treated with Diclo, Diclo + Doc and Diclo + 5-FU were examined using Glucose Assay Kit (ScienCell, 8418, USA). For the assay, cells were seeded at 1 × 10^4^ cells/well in 96-well plate in glucose-free medium and incubated for 48 h. At the end of the incubation period, the medium was collected for analysis. The method was conducted according to the kit protocol, which is based on the glucose oxidase-catalyzed oxidation of glucose and the absorbance intensity of the reaction product was measured at 570 nm.

### 2.5. Measurement of lactate release

Lactate release was measured in MCF-7 cells treated with Diclo, Diclo + Doc, and Diclo + 5-FU using a Lactate Assay Kit (Cell BioLabs, 5012, USA) to evaluate changes in glycolysis. The samples were prepared following the same procedure as used for glucose uptake measurement. After centrifugation at 10,000 rpm for 5 min, the supernatant was collected and used for analysis. Lactate content was determined based on the oxidation of lactate to pyruvate and hydrogen peroxide by lactate oxidase. The absorbance intensity at 570 nm was measured.

### 2.6. Determination of TPI levels in MCF-7 cell

To evaluate glucose metabolism at the molecular level, TPI, one of the key enzymes of glycolysis, was analyzed. For this purpose, cells treated with Diclo, Diclo + Doc, and Diclo + 5-FU (5, 10, and 15 μM) for 48 h, as well as untreated cells, were lysed. TPI levels were then measured using the ELISA method according to the kit protocols (MyBioSource, MBS7204977, USA).

### 2.7. Measurement of LDH activity

In addition to glucose, lactate and TPI analyses, the activity of LDH enzyme, which catalyzes the final step of glycolysis, was examined to evaluate the effects of the treatments on glycolysis. LDH activity was measured in MCF-7 cells treated with Diclo, Diclo + Doc, and Diclo + 5-FU, and control groups, using the LDH Activity Colorimetric Assay Kit (Elabscience, E-BC-K046-M, USA) following the kit protocol.

### 2.8. Statistical analysis

The software program GraphPad Prism 10.3.0 was used for statistical analysis. Significance was tested using one-way analysis of variance (ANOVA), and for cases where significant differences were observed, group differences were identified using the Tukey HSD Post Hoc Test. All tests were conducted at a significance level of p < 0.05.

## 3. Results

### 3.1. Effect of diclo and combination treatments on cell viability in MCF-7 cells

**Figure 1:**
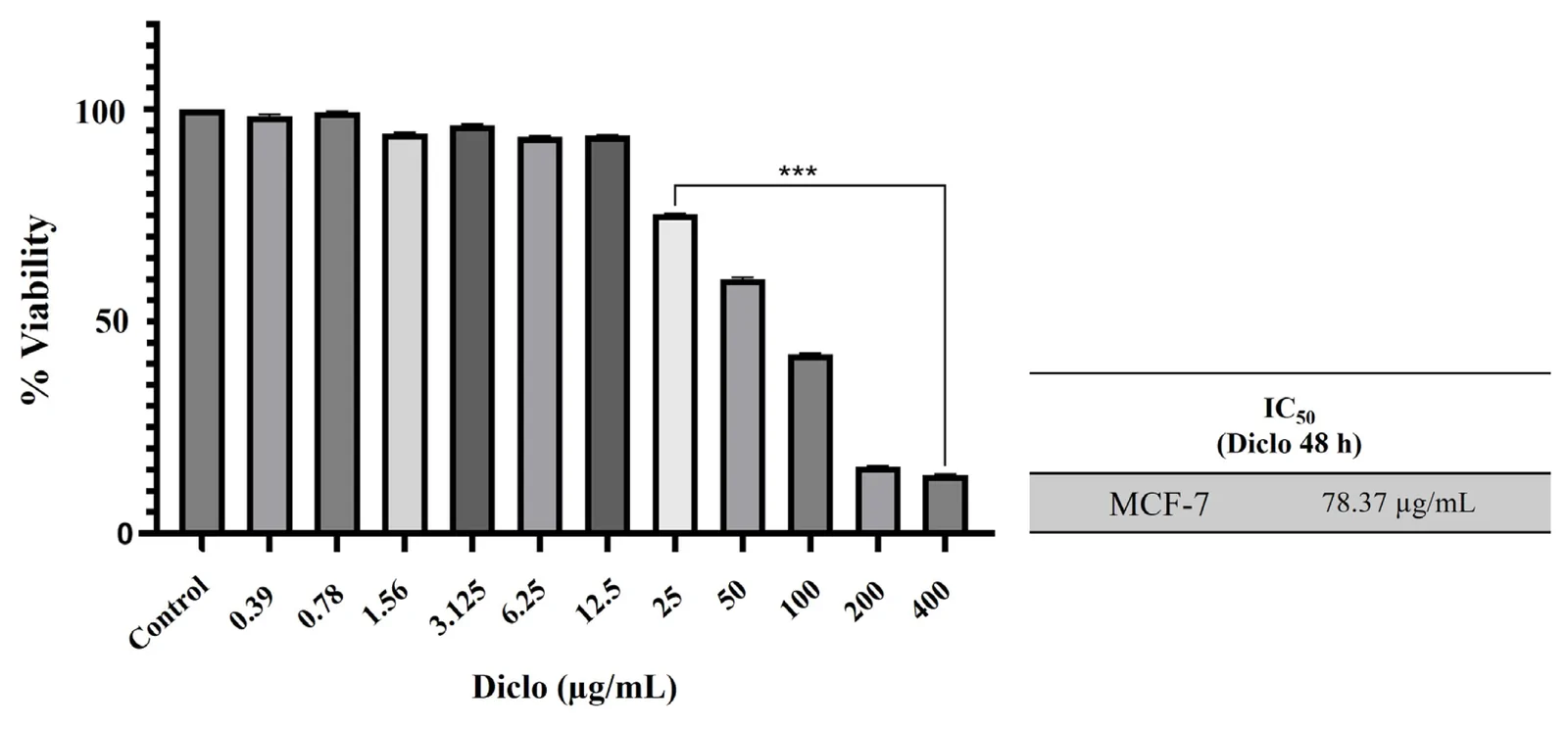
Sulforhodamine B (SRB) viability assay results (left) and IC_50_ value (right) following 48 h Diclofenac (Diclo) treatment in MCF-7 cells. *Denotes differences from the control group that are statistically significant. *** (p < 0.001). Every experiment was conducted in triplicate, and the results are shown as mean ± SD (n = 3). SD, standard deviation.

MCF-7 cells treated with Diclo for 48 h showed a dose-dependent reduction in cell viability (Figure 1). The IC_50_ value was determined to be 78.37 μg/mL, and three different Diclo concentrations (25, 50, and 100 μg/mL), including the IC_50_ value, were selected for combination experiments (Figure 1). The effects on cell viability were further investigated by combining Diclo with various doses of Doc (0.17–11.3 μg/mL) and 5-FU (0.35–22.5 μg/mL), both of which are effective chemotherapeutic agents for breast cancer treatment. This approach aimed to evaluate whether the efficacy of commonly used chemotherapy drugs could be enhanced through combination with Diclo.

**Figure 2:**
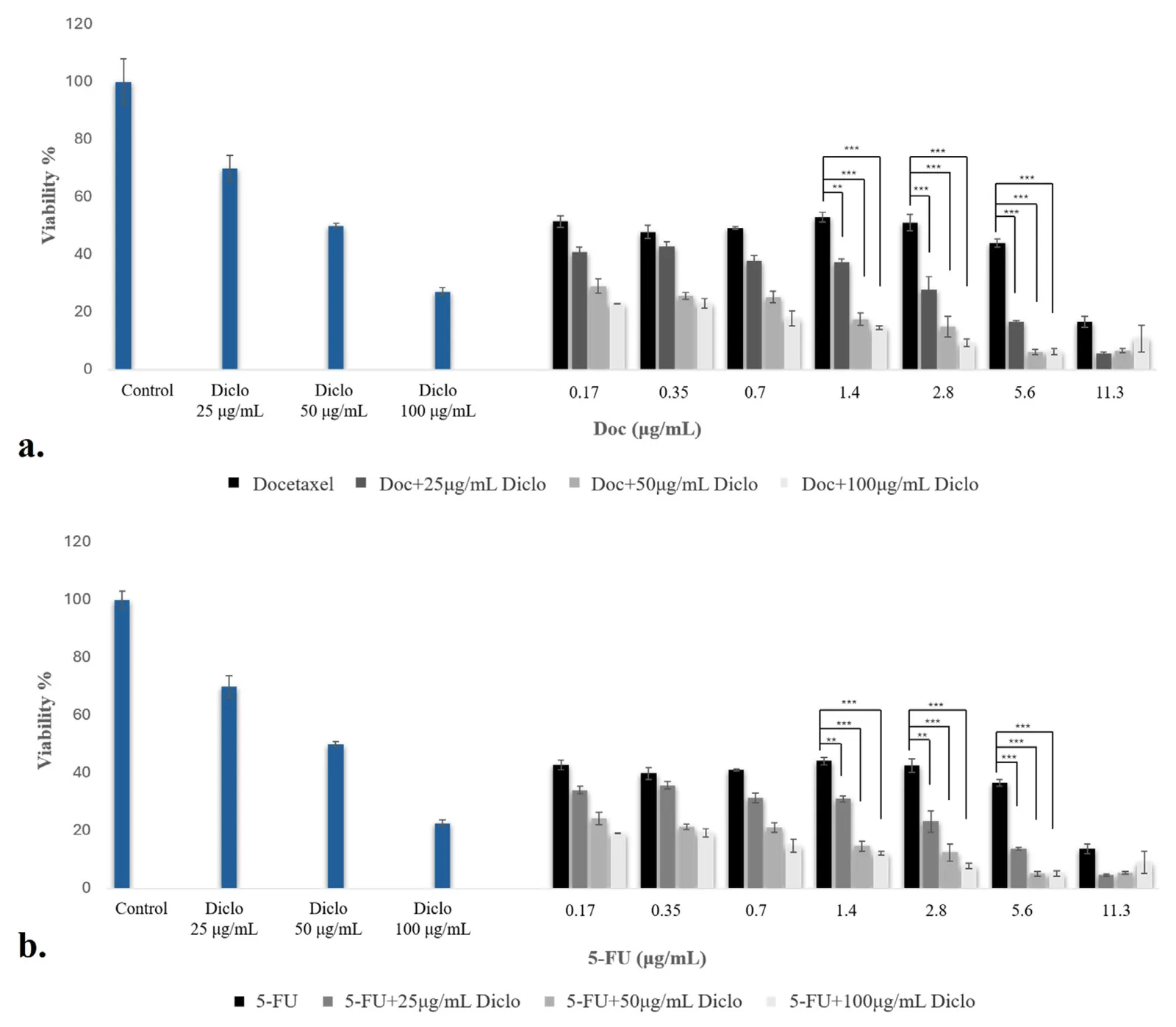
Viability of MCF-7 cells after treatment of 50 and 100 μg/mL Diclofenac (Diclo) and different doses (0.17–11.3 μg/mL) of Docetaxel (Doc) **(a)** and 5-Fluorouracil (5-FU) **(b)** alone or in combination for 48 h measured by the Sulforhodamine B (SRB) viability assay. *Denotes statistically significant differences in comparison with Doc and 5-FU alone. ** (p < 0.01); *** (p < 0.001). All experiments were triplicated, and the data are presented as mean ± SD (n = 3). SD, standard deviation.

The combination of Diclo with Doc at concentrations ranging from 1.4 to 5.6 μg/mL was found to be significantly more effective than either Doc or Diclo alone in reducing cell viability (p < 0.01 and p < 0.001) (Figure 2a). Similarly, the combination of 5-FU and Diclo led to a greater reduction in cell viability compared to their individual treatments. When 5-FU and Diclo were applied together at concentrations between 2.8 and 11.25 μg/mL, the combination induced up to six times higher cytotoxicity compared to single-agent treatments (p < 0.001) (Figure 2b).

### 3.2. Effect of diclo and combination treatments on glucose uptake and lactate release

**Figure 3:**
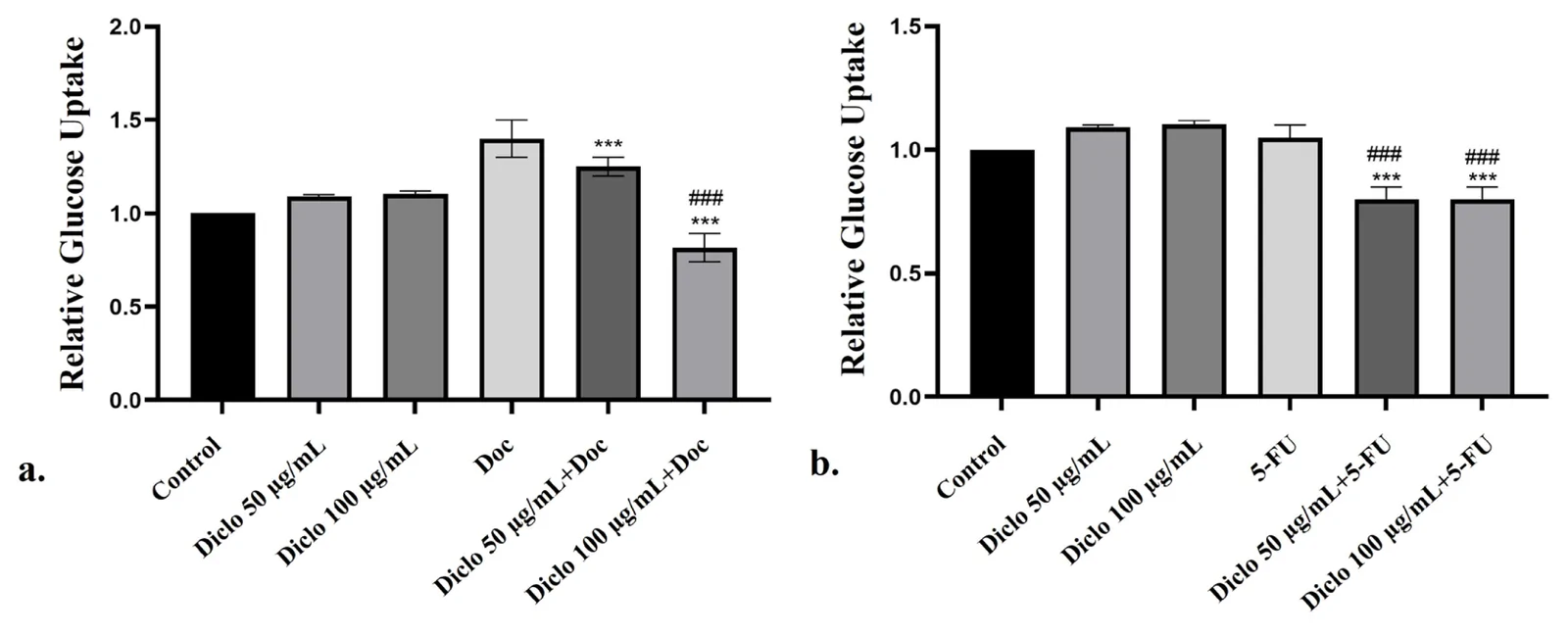
Changes in glucose uptake in MCF-7 cells following 48 h treatment with 50 μg/mL and 100 μg/mL of Diclofenac (Diclo), Diclo and 2.8 μg/mL of Docetaxel (Doc) **(a)**, Diclo and 5.6 μg/mL of 5-Fluorouracil (5-FU) **(b)**. *Denotes statistically significant differences in comparison with Diclo alone (50 and 100 μg/mL). *** (p < 0.001). # Indicates differences that are statistically significant when compared to Doc and 5-FU alone. ### (p < 0.001). All experiments were triplicated, and the data are presented as mean ± SD (n = 3). SD, standard deviation.

**Figure 4:**
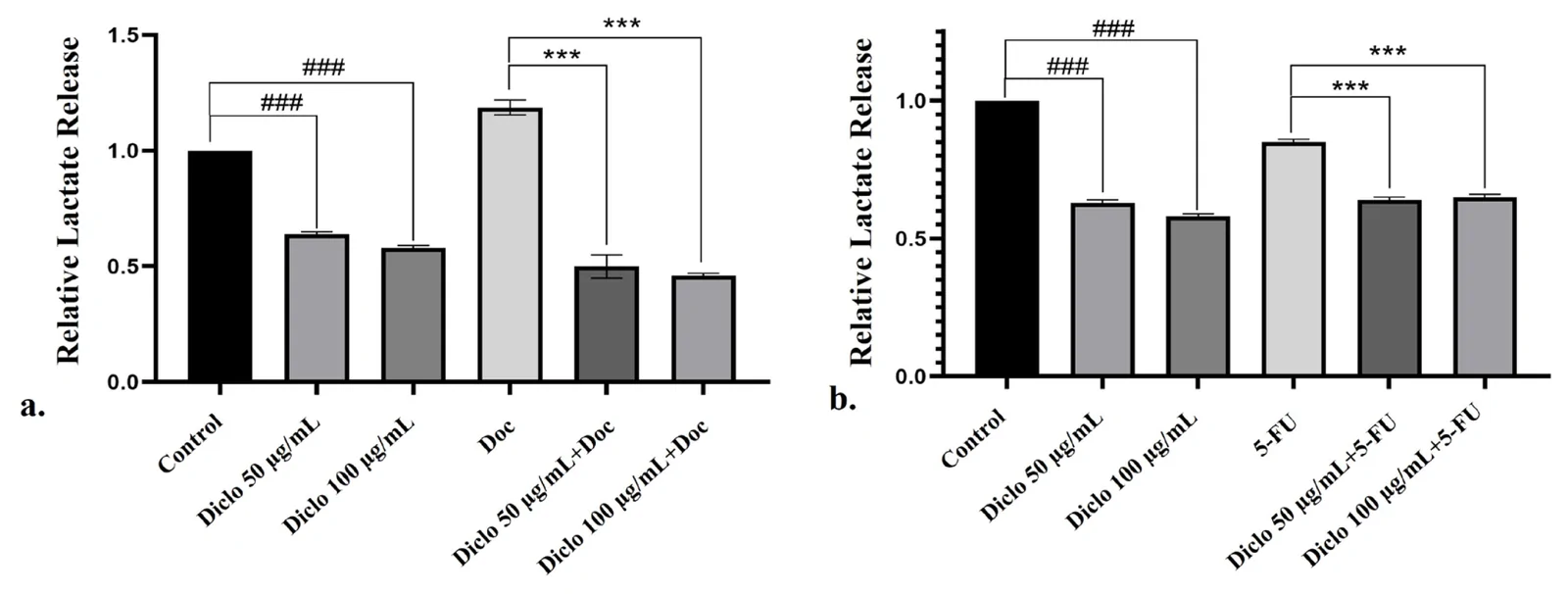
Changes in lactate release in MCF-7 cells following 48 h treatment with 50 μg/mL and 100 μg/mL of Diclofenac (Diclo), Diclo and 2.8 μg/mL of Docetaxel (Doc) **(a)**, Diclo and 5.6 μg/mL of 5-Fluorouracil (5-FU) **(b)**. # and *Denotes statistically significant differences in comparison with control, and chemotherapeutics alone (Doc and 5-FU), respectively. ### and *** (p < 0.001). All experiments were triplicated, and the data are presented as mean ± SD (n = 3). SD, standard deviation.

The effective doses were selected according to the viability analysis results and it was evaluated how Diclo alone and in combination with Doc and 5-FU affected the glycolytic capacity in MCF-7 cells. Glucose uptake was measured in cells treated with Diclo at 50 μg/mL and 100 μg/mL, in combination with Doc (2.8 μg/mL) and 5-FU (5.6 μg/mL). In addition to glucose uptake, lactate release was determined to evaluate changes in glycolytic capacity. While single-agent treatments of Diclo and chemotherapeutics did not induce significant changes in glucose uptake, the combination therapies of Diclo with Doc (Diclo + Doc) and Diclo with 5-FU (Diclo + 5-FU) significantly reduced glucose uptake in MCF-7 cells (p < 0.001) (Figure 3a., 3b). Similarly, lactate release was observed to decrease following the combination therapies (Diclo + Doc and Diclo + 5-FU). But, this decrease appears to be due to the effect of Diclo alone. Diclo alone also demonstrated a significant reduction in lactate secretion, highlighting its potential impact on glycolytic activity (p < 0.001.) (Figure 4a., 4b).

### 3.3. Changes in TPI levels after diclo, diclo+doc and diclo+5-FU treatments

**Figure 5:**
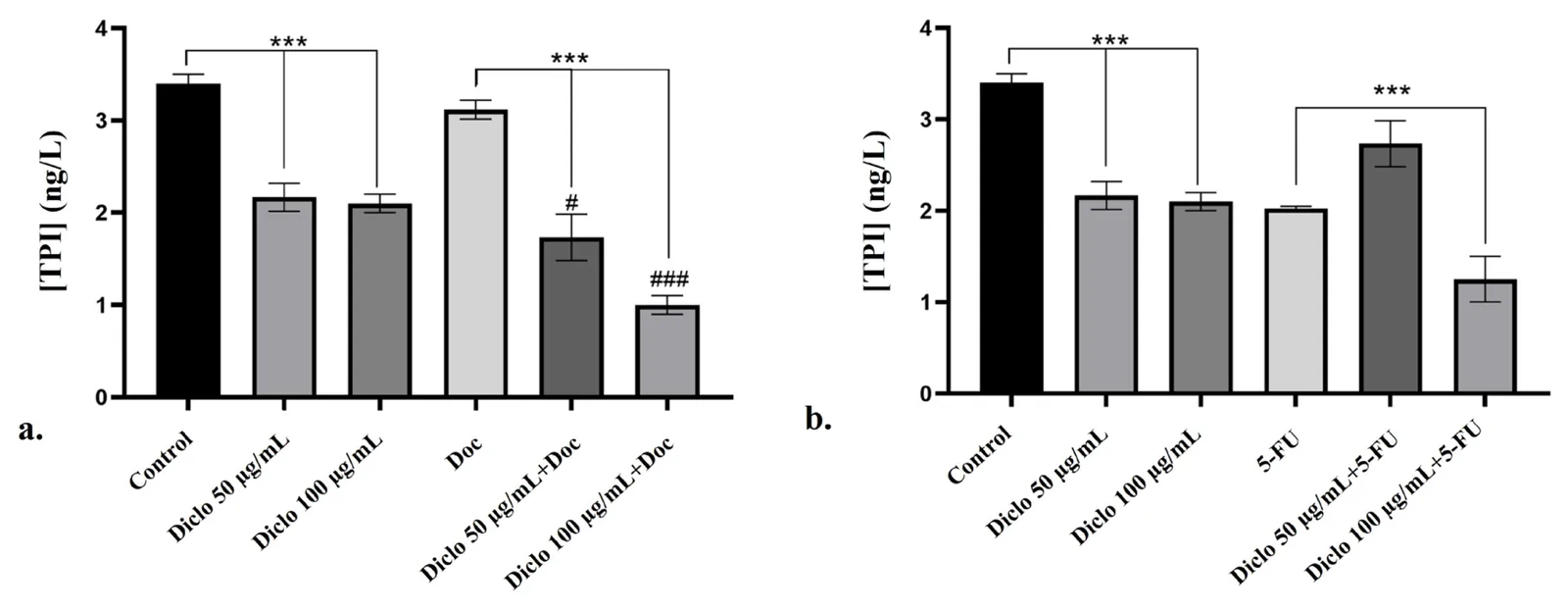
Changes in Triosephosphate Isomerase (TPI) level in MCF-7 cells following 48 h treatment with 50 μg/mL and 100 μg/mL of Diclofenac (Diclo), Diclo and 2.8 μg/mL of Docetaxel (Doc) **(a)**, Diclo and 5.6 μg/mL of 5-Fluorouracil (5-FU) **(b)**. *Denotes statistically significant differences in comparison with control, and chemotherapeutics alone (Doc and 5-FU), respectively. *** (p < 0.001). # Denotes statistically significant differences in comparison with Diclo alone (50 and 100 μg/mL). # (p < 0.05), ### (p < 0.001). All experiments were triplicated, and the data are presented as mean ± SD (n = 3). SD, standard deviation.

The effects of Diclo, alone and in combination therapies, on the levels of TPI, a key glycolytic enzyme, were evaluated. Treatment with Diclo alone at 50 and 100 μg/mL resulted in a similar reduction in TPI levels (p < 0.001). Doc alone did not significantly alter TPI level. However, combination of Doc and Diclo had an enhanced effect, leading to a further decrease in TPI levels. Notably, Diclo 100 μg/mL combined with Doc resulted in the most pronounced reduction in TPI (Figure 5a). Similarly, the combination of Diclo 100 μg/mL and 5-FU was also seen to cause a significant decrease in TPI levels compared to the chemotherapeutic drug alone (p < 0.001) (Figure 5b).

### 3.4. Changes in LDH activity following diclo and combination therapies

**Figure 6:**
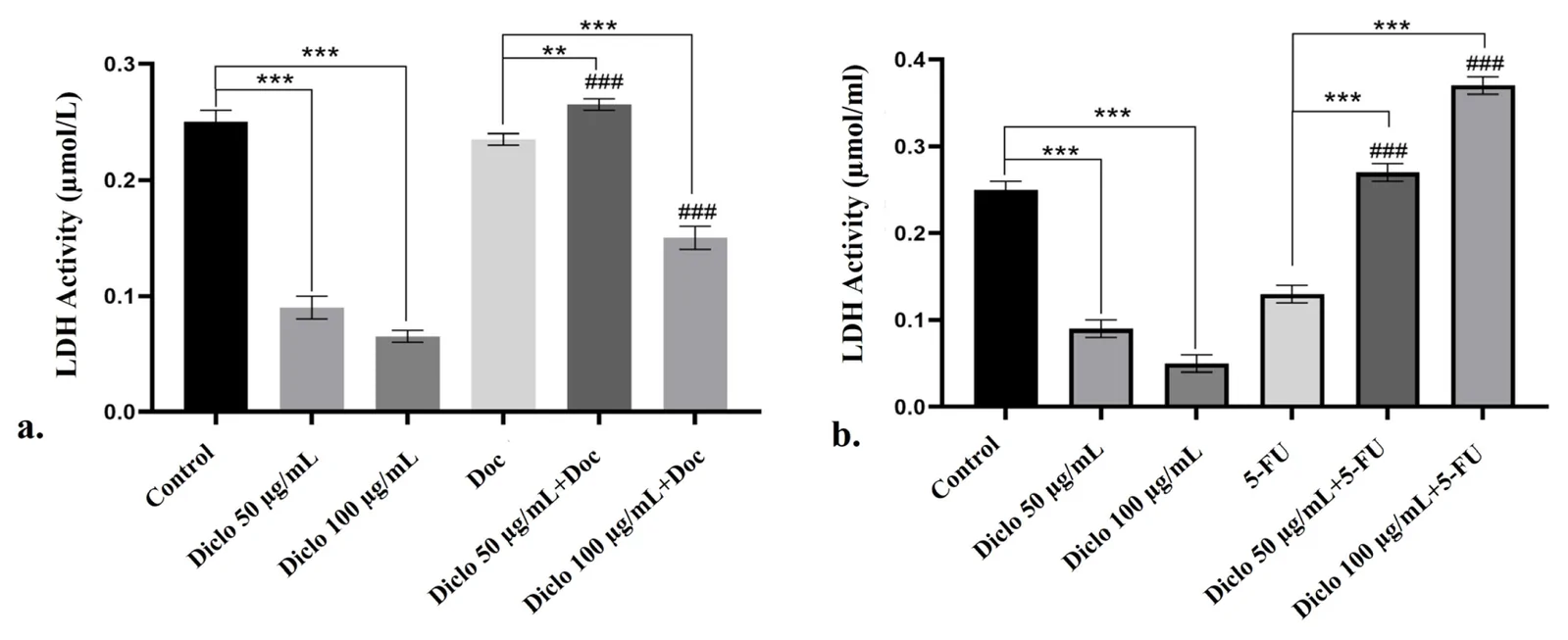
Changes in Lactate Dehydrogenase (LDH) activity in MCF-7 cells following 48 h treatment with 50 μg/mL and 100 μg/mL of Diclofenac (Diclo), Diclo and 2.8 μg/mL of Docetaxel (Doc) **(a)**, Diclo and 5.6 μg/mL of 5-Fluorouracil (5-FU) **(b)**. *Denotes statistically significant differences in comparison with control, and chemotherapeutics alone (Doc and 5-FU), respectively. *** (p < 0.001). # Denotes statistically significant differences in comparison with Diclo alone (50 and 100 μg/mL). # (p < 0.05), ### (p < 0.001). All experiments were triplicated, and the data are presented as mean ± SD (n = 3). SD, standard deviation.

In addition to evaluating TPI level, the activity of another glycolytic enzyme, LDH, responsible for lactate production, was assessed to further investigate glycolysis. Treatment with Diclo at 50 and 100 μg/mL concentrations resulted in a significant reduction in LDH activity (p < 0.001). Notably, the combination of Diclo (100 μg/mL) with Doc led to a further reduction in LDH activity relative to Doc alone. In contrast, other combination treatments involving Doc or 5-FU resulted in a significant increase in LDH activity (p < 0.001). Also, it is noteworthy that combination therapies cause an increase in LDH activity compared to Diclo treatment alone (p < 0.001) (Figure 6a., 6b).

## 4. Discussion

Diclo is a widely prescribed NSAID used to treat inflammation, pain, and fever. Beyond its analgesic and antipyretic properties, Diclo has demonstrated anticancer activity by inducing apoptosis, affecting the cell cycle, and preventing tumor growth in various cancers, including colorectal, neuroblastoma, ovarian, fibrosarcoma, pancreatic, and breast cancers. Additionally, Diclo has been associated with reduced metastasis risk in nonsmall cell lung cancer [9,10,19–22]. Consistent with the literature, our study showed that Diclo exerted a dose-dependent antiproliferative effect on MCF-7 breast cancer cells (Figure 1).

The repurposing of Diclo in cancer therapy has gained attention due to its potential to enhance treatment efficacy, reduce toxicity, and overcome resistance [23]. Combinations of Diclo with other agents have yielded promising results. For example, Diclo combined with Metformin in hamster fibrosarcoma models displayed a synergistic inhibitory effect on tumor growth [24]. Similarly, Diclo combined with 5-FU increased cancer cell death in cervical and prostate cancer [12]. In lung cancer, Diclo combined with docosahexaenoic acid (DHA) inhibited cell viability more effectively than individual treatments, suggesting potential chemopreventive applications [11]. Our study also showed that the combination of Diclo with Doc and 5-FU was more effective in reducing breast cancer cell viability than Diclo monotherapy (Figure 2). These results highlight an important strategy for achieving more efficient therapeutic effects at potentially lower drug concentrations.

Emerging research shows that Diclo’s influence on cancer metabolism, particularly glycolysis, which is frequently upregulated in cancer cells through the Warburg Effect [5,6]. Cancer cells depend on glycolysis to sustain energy demands and proliferation, making it a promising therapeutic target. Here, for the first time, we demonstrated that Diclo combined with Doc and 5-FU reduced glucose uptake and lactate release in MCF-7 cells (Figures 3, 4), suggesting impaired glycolytic flux and reduced energy production, contributing to decreased viability. While Diclo alone did not significantly alter glucose uptake, it did reduce lactate release and LDH activity (Figures 4, 6), indicating potential effects on glycolysis via lactate metabolism. On the other hand, the increased LDH activity observed with Diclo + 5-FU may reflect membrane damage rather than direct glycolytic inhibition. To harmonize these findings, LDH activity changes should be interpreted in the context of both metabolic regulation and treatment-induced cellular stress. While reduced LDH activity together with decreased lactate release supports suppressed lactate production, increased LDH activity in combinations may reflect compensatory metabolic reprogramming and/or increased enzyme release from damaged cells; thus, LDH should not be used as a standalone surrogate of glycolytic flux.

Previous studies reported Diclo-mediated disruption of Myc expression and glycolysis-related genes such as GLUT1, LDH-A, and MCT1 in various cancer cells [13,25]. Our study further identified, for the first time, that Diclo and its combinations decreased levels of TPI, a critical metabolic enzyme (Figure 5). TPI is a glycolytic enzyme, and Dihydroxyacetone phosphate (DHAP) and glyceraldehyde-3-phosphate (G3P) are interconverted by TPI. For glycolysis and gluconeogenesis to produce energy efficiently, this process is essential. Research indicates that TPI is elevated in a number of cancer types, such as colorectal, lung, and breast cancer [26–28]. Increased TPI activity contributes to enhanced glycolytic flux, providing cancer cells with ATP and biosynthetic precursors. In this way, TPI is believed to have a role in the genesis of tumors by encouraging the growth, migration, and invasion of cancer cells [29,30]. For instance, silencing TPI in lung adenocarcinoma significantly reduces cell migration, colony formation, and xenograft tumor growth [31]. So, inhibitors targeting glycolytic enzymes, including TPI, are being explored as potential anticancer therapies. Our results suggest that combining TPI inhibition with existing chemotherapeutics such as Doc and 5-FU may enhance treatment efficacy. In one of our recent studies, we investigated the effect of resveratrol, a natural polyphenolic compound, on TPI in breast cancer. Our findings revealed that treatment with resveratrol led to a decrease in TPI levels, reduced the glycolysis rate, and potentially induced cancer cell death via inhibiting mitogen-activated protein kinase (MAPK) pathway [32]. By reducing TPI, Diclo may contribute to glycolysis inhibition and cancer cell growth suppression, particularly in ER/PR-positive luminal A breast cancers.

MCF-7 cell line is a well-established luminal A model (ER+/PR+/HER2−) widely used to study hormone receptor–positive breast cancer biology and therapeutic responses. Considering that COX-2/PGE2 signaling is linked to estrogen biosynthesis via aromatase induction in the breast tumor microenvironment, COX inhibition by Diclo may indirectly attenuate ER-driven proliferation [33]. In addition to COX-dependent actions, Diclo has also been reported to exert COX-independent antitumor effects by targeting Myc and modulating glucose metabolism, as mentioned above, providing a mechanistic rationale for evaluating glycolytic readouts (glucose uptake, lactate release) and metabolic enzyme levels in this model [13].

Inhibiting TPI could also impact other metabolic pathways, including the pentose phosphate pathway and fatty acid synthesis [34,35]. The decline in TPI levels, acting on several metabolic pathways, may reflect direct modulation of metabolic enzyme expression, or broader stress responses that alter protein turnover. Because our study did not measure TPI transcription, protein stability, or enzyme activity directly, these mechanistic possibilities should be addressed in future studies.

In addition, studies in TNBC MDA-MB-231 cells demonstrated that Diclo downregulates GLUT1 and Myc, reduces hexokinase (HK) activity, and suppresses glycolysis via the Myc pathway [14]. While MDA-MB-231 cells with TNBC characteristics primarily rely on aerobic glycolysis as their main energy pathway, MCF-7 breast cancer cells preferentially utilize oxidative phosphorylation under normoxic conditions [17]. Our observations showed that, consistent with the metabolic phenotype, Diclo alone had limited impact on glucose uptake in MCF-7 cells. However, combination therapies notably suppressed both glucose uptake and lactate secretion, supporting that antiglycolytic strategies may vary across cancer subtypes depending on metabolic preferences.

In conclusion, our study is the first to demonstrate the effects of Diclo, alone and combined with Doc and 5-FU, on glycolytic metabolism in breast cancer. By disrupting glycolysis and reducing TPI levels, these combinations impaired glucose metabolism and decreased cell viability in ER/PR-positive MCF-7 cells. These results underscore Diclo’s potential as a metabolic modulator in cancer therapy and highlight the importance of drug repurposing strategies. Further studies should explore the detailed mechanisms of Diclo’s metabolic effects and its potential synergy with other targeted therapies.

Despite providing valuable insights, the present work has several limitations, including the restriction to a single luminal-type breast cancer cell line (MCF-7), which limits generalizability across heterogeneous breast cancer subtypes (e.g., HER2-positive and TNBC). In addition, we did not include in vivo experiments. Therefore, future work should validate these findings across multiple breast cancer subtypes and incorporate xenograft models, additional metabolic flux assays, and larger-scale protein/gene panels to confirm pathway-level mechanisms.

## Footnotes

1 World Health Organization (2024). Global cancer burden growing, amidst mounting need for services [online]. Website https://www.who.int/news/item/01-02-2024-global-cancer-burden-growing--amidst-mounting-need-for-services [accessed 15 July 2025].

2 Iqvıa (2020). Market Segmentation [online]. Website https://www.iqvia.com/insights [accessed 10 July 2025]

## References

[1] Ashraf MA Phytochemicals as potential anticancer drugs: time to ponder nature’s bounty BioMed Research International 2020 2020 8602879 10.1155/2020/8602879 32076618 PMC7013350

[2] Bizuayehu HM Ahmed KY Kibret GD Dadi AF Belachew SA Global Disparities of Cancer and Its Projected Burden in 2050 JAMA Netw Open 2024 7 11 e2443198 10.1001/jamanetworkopen.2024.43198 39499513 PMC11539015

[3] Frezza C Metabolism and cancer: the future is now British Journal of Cancer 2020 122 2 133 135 10.1038/s41416-019-0667-3 31819199 PMC6978144

[4] Hanahan D Weinberg RA Hallmarks of cancer: the next generation Cell 2011 144 5 646 674 10.1016/j.cell.2011.02.013 21376230

[5] Warburg O On the origin of cancer cells Science 1956 123 3191 309 314 10.1126/science.123.3191.309 13298683

[6] Warburg O On respiratory impairment in cancer cells Science 1956 124 3215 269 270 10.1126/science.124.3215.267 13351639

[7] Cha YI DuBois RN NSAIDs and cancer prevention: targets downstream of COX-2 Annual Review of Medicine 2007 58 239 252 10.1146/annurev.med.57.121304.131253 17100552

[8] Ghanghas P Jain S Rana C Sanyal SN Chemopreventive action of non-steroidal anti-inflammatory drugs on the inflammatory pathways in colon cancer Biomed Pharmacother 2016 78 239 247 10.1016/j.biopha.2016.01.024 26898448

[9] Pantziarka P Sukhatme V Bouche G Meheus L Sukhatme VP Repurposing drugs in oncology (ReDO)-diclofenac as an anti-cancer agent Ecancermedicalscience 2016 10 610 10.3332/ecancer.2016.610 26823679 PMC4720497

[10] Amanullah A Upadhyay A Dhiman R Singh S Kumar A Development and challenges of diclofenac-based novel therapeutics: targeting cancer and complex diseases Cancers(Basel) 2022 14 18 4385 10.3390/cancers14184385 36139546 PMC9496891

[11] Poku RA Jones KJ Van Baren M Alan JK Amissah F Diclofenac enhances docosahexaenoic acid-induced apoptosis in vitro in lung cancer cells Cancers(Basel) 2020 12 9 2683 10.3390/cancers12092683 32962236 PMC7564004

[12] Choi S Kim S Park J Lee SE Kim C Diclofenac: a nonsteroidal anti-inflammatory drug inducing cancer cell death by inhibiting microtubule polymerization and autophagy flux Antioxidants(Basel) 2022 11 5 1009 10.3390/antiox11051009 35624874 PMC9138099

[13] Gottfried E Lang SA Renner K Bosserhoff A Gronwald W New aspects of an old drug—diclofenac targets MYC and glucose metabolism in tumor cells PLoS One 2013 8 7 e66987 10.1371/journal.pone.0066987 23874405 PMC3706586

[14] Yang L Li J Li Y Zhou Y Wang Z Diclofenac impairs the proliferation and glucose metabolism of triple-negative breast cancer cells by targeting the c-Myc pathway Experimental and Therapeutic Medicine 2021 21 6 584 10.3892/etm.2021.10016 33850556 PMC8027724

[15] Çetin Ö İlaç yeniden konumlandırma yaklaşımı ile alfa tübülin asetilasyonunu arttırabilecek bileşiklerin araştırılması Yüksek Lisans Tezi, Hacettepe Üniversitesi, Sağlık Bilimleri Enstitüsü Ankara, Türkiye 2020 (in Turkish)

[16] Lee AV Oesterreich S Davidson NE MCF-7 Cells—Changing the Course of Breast Cancer Research and Care for 45 Years Journal of the National Cancer Institute 2015 107 7 djv073 10.1093/jnci/djv073 25828948

[17] Avagliano A Ruocco MR Aliotta F Belviso I Accurso A Mitochondrial Flexibility of Breast Cancers: A Growth Advantage and a Therapeutic Opportunity Cells 2019 8 5 401 10.3390/cells8050401 31052256 PMC6562467

[18] Ari F Erkisa M Pekel G Erturk E Buyukkoroglu G Anticancer potential of albumin bound Wnt/β-catenin pathway inhibitor niclosamide in breast cancer cell Chemistry Select 2021 6 29 7463 7475 10.1002/slct.202100819

[19] Hixson LJ Alberts DS Krutzsch M Einsphar J Brendel K Antiproliferative effect of nonsteroidal antiinflammatory drugs against human colon cancer cells Cancer Epidemiol Biomarkers Prev 1994 3 5 433 438 7920212

[20] Johnsen JI Lindskog M Ponthan F Pettersen I Elfman L Cyclooxygenase-2 is expressed in neuroblastoma, and nonsteroidal anti-inflammatory drugs induce apoptosis and inhibit tumor growth in vivo Cancer Research 2004 64 20 7210 7215 10.1158/0008-5472.CAN-04-1795 15492235

[21] Forget P Machiels JP Coulie PG Berliere M Poncelet AJ Neutrophil:lymphocyte ratio and intraoperative use of ketorolac or diclofenac are prognostic factors in patients undergoing cancer surgery Annals of Surgical Oncology 2013 20 3 650 660 10.1245/s10434-013-3136-x 23884751

[22] Valle BL D’Souza T Becker KG Wood WH Zhang Y Non-steroidal anti-inflammatory drugs decrease E2F1 expression and inhibit cell growth in ovarian cancer cells PLoS One 2013 8 4 e61836 10.1371/journal.pone.0061836 23637916 PMC3634839

[23] Correia AS Gärtner F Vale N Drug combination and repurposing for cancer therapy: the example of breast cancer Heliyon 2021 7 1 e05948 10.1016/j.heliyon.2021.e05948 33490692 PMC7810770

[24] Popović DJ Popović KJ Miljković D Popović JK Lalošević D Diclofenac and metformin synergistic dose-dependent inhibition of hamster fibrosarcoma, rescued with mebendazole Biomed Pharmacother 2023 167 115528 10.1016/j.biopha.2023.115528 37738800

[25] Chirasani SR Leukel P Gottfried E Hochrein J Stadler K Diclofenac inhibits lactate formation and efficiently counteracts local immune suppression in a murine glioma model International Journal of Cancer 2013 132 4 843 853 10.1002/ijc.27712 22752934

[26] Zhang XZ Xiao ZF Li C Xiao ZQ Yang F Triosephosphate isomerase and peroxiredoxin 6, two novel serum markers for human lung squamous cell carcinoma Cancer Science 2009 100 12 2396 2401 10.1111/j.1349-7006.2009.01314.x 19737146 PMC11159988

[27] Lone SN Maqbool R Parray FQ Ul Hussain M Triose-phosphate isomerase is a novel target of miR-22 and miR-28, with implications in tumorigenesis Journal of Cellular Physiology 2018 233 11 8919 8929 10.1002/jcp.26821 29856481

[28] Jin X Wang D Lei M Guo Y Cui Y TPI1 activates the PI3K/AKT/mTOR signaling pathway to induce breast cancer progression by stabilizing CDCA5 Journal of Translational Medicine 2022 20 1 191 10.1186/s12967-022-03370-2 35509067 PMC9066866

[29] Chen T Huang Z Tian Y Lin B He R Clinical significance and prognostic value of Triosephosphate isomerase expression in gastric cancer Medicine 2017 96 19 e6770 10.1097/MD.0000000000006865 28489783 PMC5428617

[30] Chen T Huang Z Tian Y Wang H Ouyang P Role of triosephosphate isomerase and downstream functional genes on gastric cancer Oncology Reports 2017 38 3 1822 1832 10.3892/or.2017.5846 28737830

[31] Liu P Sun SJ Ai YJ Feng X Zheng YM Elevated nuclear localization of glycolytic enzyme TPI1 promotes lung adenocarcinoma and enhances chemoresistance Cell Death and Disease 2022 13 3 205 10.1038/s41419-022-04655-6 35246510 PMC8897412

[32] Tuna G Akgün O Arı F Triosephosphate Isomerase Inhibition by Resveratrol: A New Mechanism of Anti-Glycolysis in Breast Cancer Molecular Biology 2024 58 6 1219 1229 10.1134/s0026893324700663

[33] Davies G Martin LA Sacks N Dowsett M Cyclooxygenase-2 (COX-2), aromatase and breast cancer:a possible role for COX-2 inhibitors in breast cancer chemoprevention Annals of Oncology 2002 13 5 669 678 10.1093/annonc/mdf125 12075734

[34] Maquat L Chilcote R Ryan P Human triosephosphate isomerase cDNA and protein structure. Studies of triosephosphate isomerase deficiency in man The Journal of Biological Chemistry 1985 260 1 37 48 10.1016/S0021-9258(19)83687-6 2579079

[35] Orosz F Oláh J Ovádi J Triosephosphate isomerase deficiency: facts and doubts IUBMB Life 2006 58 12 703 715 10.1080/15216540601115960 17424909

